# Cardioprotective effects of the electrolyte solution sterofundin and the possible underlying mechanisms

**DOI:** 10.3389/fphar.2024.1449831

**Published:** 2025-01-03

**Authors:** Min Chen, Yingying Xiao, Jijian Zheng, Peibin Zhao, Lin Cheng, Chuan Jiang, Sixie Zheng, Zheng Wang, Sijuan Sun, Lincai Ye, Guozhen Chen, Hao Zhang, Yanhui Huang

**Affiliations:** ^1^ Children’s Heart Center, Institute of Cardiovascular Development and Translational Medicine, The Second Affiliated Hospital and Yuying Children’s Hospital, Wenzhou Medical University, Wenzhou, China; ^2^ Department of Anesthesiology, Shanghai Children’s Medical Center, Shanghai Jiao Tong University School of Medicine, Shanghai, China; ^3^ Department of Thoracic and Cardiovascular Surgery, Shanghai Children’s Hospital, Shanghai Jiao Tong University, School of Medicine, Shanghai, China; ^4^ Department of Thoracic and Cardiovascular Surgery, Shanghai Children’s Medical Center, Shanghai Jiao Tong University School of Medicine, Shanghai, China; ^5^ Department of Pediatric Intensive Care Unit, Shanghai Children’s Medical Center, Shanghai Jiao Tong University School of Medicine, Shanghai, China; ^6^ Shanghai Institute for Pediatric Congenital Heart Disease, Shanghai Children’s Medical Center, School of Medicine Shanghai Jiao Tong University, Shanghai, China; ^7^ Department of Cardiology, Shanghai Children’s Medical Center, Shanghai Jiao Tong University School of Medicine, Shanghai, China

**Keywords:** sterofundin, electrolyte solutions, myocardium infarction, intensive care, autophagy

## Abstract

**Background:**

Sterofundin (SF) is one of the most widely used electrolyte solutions in almost all areas of medicine, with particular importance in intensive care. It provides powerful correction of acid-base imbalances, ion fluctuations, and impaired energy metabolism, which are the three most important characteristics after myocardial infarction (MI). However, whether and how SF protects the heart from post-MI damage are largely unknown.

**Methods and Results:**

Pretreating mice with SF before MI surgery reduced the number of reactive oxygen species (ROS)-positive and TUNEL-positive cells. As a result, the infarcted area cardiac fibrosis in the MI mice was reduced and cardiac performance in the MI mice improved. Moreover, RNA-seq analysis demonstrated that SF caused the gene expression profile of MI mice to shift toward that of sham mice, with a significant decrease in apoptosis-, ROS-, and inflammation-associated gene enrichment. RNA-seq analysis also demonstrated that SF induced the upregulation of autophagy-associated gene enrichment. Western blotting confirmed the RNA-seq analysis results, showing that SF induced the upregulation of an autophagic flux. When the autophagic flux was blocked with the autophagy inhibitor 3-methyladenine, the protective effect of SF was reduced.

**Conclusion:**

SF protects the heart from post-MI damage, and one of the underlying mechanisms could be its autophagy modifications. This study is the first to reveal a previously unrecognized role of electrolyte solutions in post-MI intensive care.

## Highlights

• A possible role of an electrolyte solution in autophagy modification is revealed for the first time, enhancing our understanding of electrolyte solutions in intensive care.
• The electrolyte solution Sterofundin provides MI protection.

## 1 Introduction

Sterofundin (SF), one of the most widely used electrolyte solutions in clinical therapies, is particular important in intensive care (Curran et al., 2021; Zadák et al., 2010; László et al., 2019). The main function of electrolyte solutions in clinical treatment is to maintain the stability of the internal environment, including the acid-base balance, physiological potassium/calcium ratio, and proper osmotic pressure (Curran et al., 2021; Zadák et al., 2010; László et al., 2019; Kaufman et al., 2023). Physiologically, the potassium/calcium ratio is largely dependent on the acid-base balance (Martinez et al., 2016; Kelly and Levine, 2013; Rylander et al., 2009). In addition to maintaining the acid-base balance, as do other electrolyte solutions, SF plays an important role in stabilizing energy metabolism due to its metabolizable organic anions (e.g., acetate, malate), which may affect oxygen consumption (Zadák et al., 2010).

In acute myocardium infarction (MI), the combination of decreased cardiac output and arterial hypoxemia produce tissue hypoxia and metabolic acidosis, which are closely correlated to mortality, making the acid-base imbalance one of the most important characteristics post-MI (Kirby and McNicol, 1966; Lazzeri et al., 2010; Widomska-Czekajska, 1976). In addition, ion fluctuations constitute the second characteristic found post-MI (Zhang et al., 2021; Afridi et al., 2010; Singh et al., 1990), and impaired energy metabolism is the third characteristic of MI (Frangogiannis, 2015; Han et al., 2023; Opie, 1975). Thus, SF’s functions are well matched to the characteristics of the post-MI state. However, whether and how SF reduces post-MI damage are largely unknown.

The main pathological changes post-MI are apoptosis and necrosis of myocardial cells, as well as inflammatory responses and the reactive oxygen species (ROS) induced by these responses (Wang et al., 2018; Akodad et al., 2020; Cai et al., 2023). If ROS and necrotic cells cannot be cleared on time, they will further cause apoptosis and necrosis of myocardial cells, forming a vicious cycle (Wang et al., 2018; Akodad et al., 2020; Cai et al., 2023). It should be highlighted that autophagy plays a major role in clearing ROS and necrotic cells post-MI, maintaining the intracellular homeostasis of myocardial cells (Zhang et al., 2022; Bravo-San Pedro et al., 2017). In addition, autophagy is closely associated with acid-base and metabolic balances (Song et al., 2018; Glick et al., 2010; Mizushima and Komatsu, 2011). It is possible that SF reduces post MI-damage via autophagy modifications. We are the first to investigate whether and how SF reduces post-MI damage. We then explored the role of autophagy in MI protection by SF.

## 2 Materials and methods

### 2.1 Statement of data availability

RNA-seq data were deposited in NCBI’s Gene Expression Omnibus database (https://www.ncbi.nlm.nih.gov/geo) with accession number GSE245917.

### 2.2 Ethics statement

**FIGURE 1:**
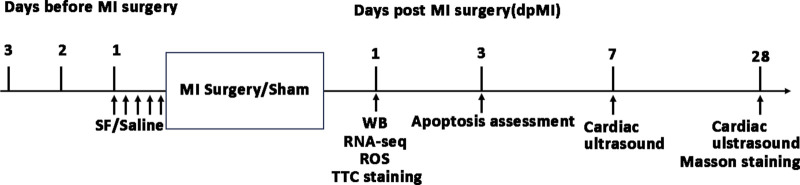
Timeline of experiments in the current study. MI: myocardium infarction; SF: Sterofundin; WB: Western blotting; ROS: reactive oxygen species.

All procedures (Figure 1) described in this study conformed to the requirements of the Helsinki Declaration and received approval from the Animal Welfare and Human Studies Committee of Shanghai Children’s Medical Center (IRB number: SCMCIRB—K2022146-1).

### 2.3 Myocardial infarction

C57/BL6 mice, male or female, 6–8 weeks old, were purchased from Xipu’er-bikai Experimental Animal Co., Ltd. (Shanghai, China). The mice were randomly divided into four groups: ① sham group, in which the chest was only opened and closed without left anterior descending coronary artery (LAD) ligation; ② MI group, with LAD ligation; ③ MI + SF group, in which 1 mL/20 g SF (B. Braun Melsungen AG Division Hospital Care 34,212, Melsungen, Germany) (the dosages were the same as in clinical use (Mishra et al., 2017)) was administered via the tail vein 24 h before MI surgery, with 0.2 mL each time for 5 times. The last injection was 1 hour before MI surgery; ④ MI + SF+3-methyladenine (3-MA, M9281, Sigma, Shanghai, China) group, in which 1 h before MI surgery, SF (1 mL/20 g) was injected through the tail vein, and 3-MA (0.3 mg/20 g, dissolved in dimethyl sulfoxide (DMSO, 472,301, Sigma, Shanghai, China) to prepare a 100 mM solution and diluted with physiological saline before use) was injected intraperitoneally. Because this injection method is not commonly used, we provided a more detail information concerning injection methods in the supplemental methods. MI surgery was performed as follows: the mice were anesthetized, intubated, and ventilated as previously described (Baehr et al., 2020). After a left lateral thoracotomy, the LAD was ligated with an 8–0 nylon suture. After ligation, the apex turned white. Then, the chest was closed and the mice were placed on a constant temperature electric blanket at 37°C until they regained autonomous movement. The mortality rate of MI surgery is zero.

### 2.4 Infarct area evaluation

One day post-MI surgery (dpMI), the time before obvious scar forming, TTC staining was used to assess the infarct area, as suggested by Guidelines for Experimental Models of Myocardial Ischemia and Infarction (Lindsey et al., 2018). For permanent coronary occlusion MI models that pass the point from ischemia to infarction, the use of Evans blue is not necessary for area-at-risk assessment, as the entire area at risk is infarcted, as stated the above guidelines (Lindsey et al., 2018). Six mice were randomly selected from each group from the remaining mice and euthanized by cervical dislocation under anesthesia. The hearts were sectioned into five parts, incubated with 1% TTC in PBS for 15 min at 37°C, and protected from light, as suggested by the manufacturer.

### 2.5 Masson’s trichrome staining

On 28 dpMI, cardiac tissues were subjected to Masson’s Trichrome Staining using Masson’s Trichrome Staining kits (C0189S, Beyotime, Shanghai, China) according to the manufacture’s suggestions. Briefly, the tissues were stained with hematoxylin, eosin, and bright green in sequence. The slides were then washed, sealed, and imaged.

### 2.6 Determination of ROS-positive cells

On 1 dpMI, six mice were randomly selected from each group from the remaining mice and euthanized by cervical dislocation under anesthesia. the hearts were subjected to a ROS assay with a ROS assay kit (S0033S, Beyotime, China). Briefly, the myocardial cells were isolated with a Langendroff perfusion system and incubated with DCFH-DA (provided in the kit) for 20 min at 37°C. After washing with PBS buffered with 10% serum three times, the ROS-positive cells were measured by flow cytometry.

### 2.7 RNA-seq analysis

On 1 dpMI, three mice were randomly selected from each group from the remaining mice and euthanized by cervical dislocation under anesthesia. The hearts were collected for total mRNA preparation and were extracted using a PureLink RNA micro scale kit (12,183,016, Life Technologies, Carlsbad, CA, United States). The mRNA was then used to generate sequencing libraries with a NEBNext^®^ Ultra RNA library prep kit (E7760, NEB, United States) following the manufacturer’s recommendations. The library was sequenced on an Illumina Novaseq platform to generate raw data, which were processed through in-house PERL scripts to produce high-quality clean data. The clean data were used for differential expression analysis, and for gene ontology (GO) and KEGG enrichment analysis. The ClusterProfiler R package was used to test for statistical enrichment.

### 2.8 Western blot

On 1 dpMI, six mice were randomly selected from each group from the remaining mice and euthanized by cervical dislocation under anesthesia. The hearts were collected for Western blot. Briefly, total proteins were extracted with RIPA lysis buffer (P0013B, Beyotime, Shanghai, China) according to the manufacturer’s instruction, separated on 10% SDS polyacrylamide gels, and transferred onto polyvinylidene fluoride membranes (Merck, Millipore, Billerica, MA, United States), which were blocked with 5% non-fat milk in tris-buffered saline with Tween 20 (TBST) for 1 h at 24°C. The membranes were incubated with anti-LC3I/II (4,108, Cell Signaling) and anti-p62 (5,114, Cell Signaling) antibodies overnight at 4°C. After washing with TBST three times, the membranes were incubated with Dylight 800-labeled affinity secondary antibodies for 1 h at 24°C and analyzed with a ChemiDoc™ imaging system (Bio-Rad). ImageJ (NIH) was used for quantitative densitometry image analysis.

### 2.9 Apoptosis evaluation

On 3 dpMI, Six to eight mice were randomly selected from each group from the remaining mice and euthanized by cervical dislocation under anesthesia. The hearts were collected for slide sectioning. After fixing with 4% paraformaldehyde, the tissue was permeated with 0.5% Triton X-100 for 15 min, blocked with 10% donkey serum for 30 min, and stained using a TdT-mediated dUTP nick end labeling (TUNEL) kit (C1082, Beyotime, China) according to the manufacturer’s instructions. After TUNEL staining, the slides were subjected to sarcomeric α-actinin (SAA) antibody (ab9465, Abcam, China) and 4′,6-diamidino-2-phenylindole (DAPI) staining.

### 2.10 Cardiac ultrasound

On 7 or 28 dpMI, six to eight mice were randomly selected from each group from the remaining mice were subjected to a cardiac performance evaluation with echocardiography. Briefly, the mice were anesthetized with 5% isoflurane and secured in the supine position. Then, pre-warmed echo gel was placed on the left thoracic area. A parasternal long-axis view of the left ventricle (LV) was obtained. The ultrasound probe (90 MHz) was turned 90° to obtain a parasternal short-axis view, and M-mode images were recorded. Ejection fraction (EF%), shortening fraction (FS%), left ventricular end systolic diameter (LVESD), and left ventricular end diastolic diameter (LVEDD) were collected.

### 2.11 Statistical analysis

Continuous data, including EF%, infarct size, LVEDD, LVESD, FS%, protein expression, and the number of TUNEL positive cells, were expressed as mean ± standard deviation. If the data were normally distributed, we used Student’s t-test, ANOVA, or Student–Newman–Keuls (SNK) test for back testing to evaluate the difference. Otherwise, we used rank sum tests. A P-value <0.05 was considered statistically significant. SAS software version 9.2 (SAS Institute Inc., Cary, NC, United States) was used for statistical analysis.

## 3 Results

### 3.1 SF pretreatment reduces ROS and cell necrosis caused by MI in the early period

**FIGURE 2:**
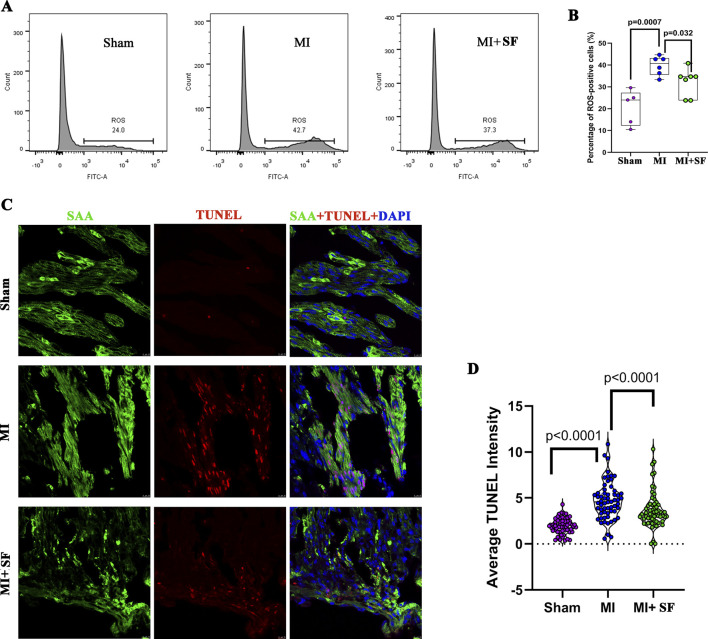
Sterofundin (SF) pretreatment reduces ROS and cell necrosis post-myocardial infarction (MI). **(A)** Representative flow cytometry results for ROS-positive cells post-MI. **(B)** Quantification of ROS-positive cells. **(C)** Representative TUNEL-positive cells. SAA (Sarcometric α actinin, marker of cardiomyocyte, green), DAPI (blue), and TUNEL (red). **(D)** Quantification of TUNEL intensity. ANOVA, SNK test.

As shown in Figures 2A,B, on 1 dpMI, flow cytometry demonstrated that the percentage of ROS-positive cells was significantly reduced in the MI + SF groups compared to the MI group. The apoptosis marker TUNEL was also significantly reduced in the MI + SF groups compared to the MI group (Figures 2C,D). These results indicated that SF pretreatment reduced ROS and cell necrosis caused by MI in the early period.

### 3.2 SF pretreatment reduces infarcted area and increases cardiac performance in the early period

**FIGURE 3:**
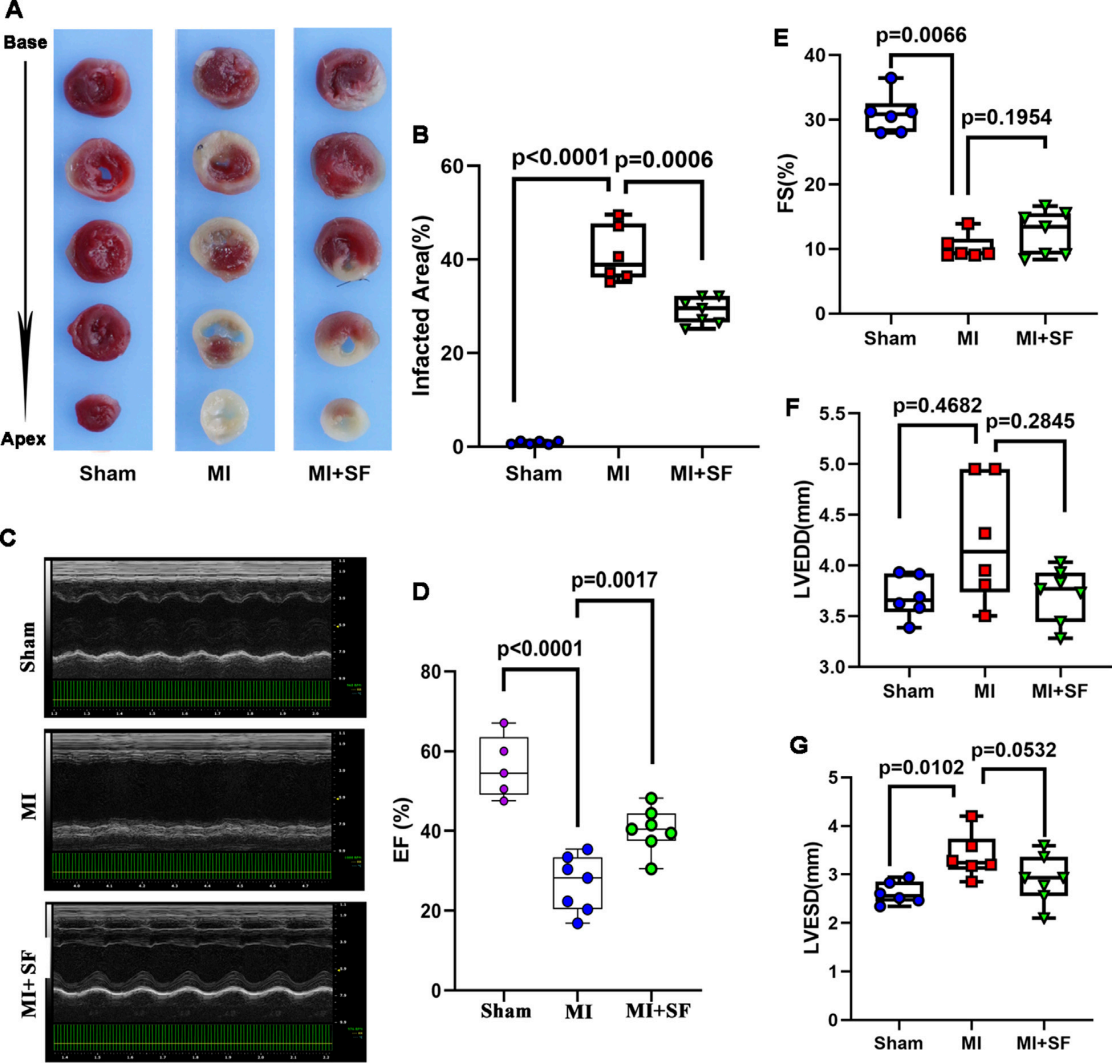
SF pretreatment reduces infarcted size and increases cardiac function in the early period. **(A)** Representative infarcted area by TTC staining. **(B)** Quantification of infarcted area (%). **(C)** Representative echocardiogram. **(D)** Quantification of EF%. **(E)** Quantification of FS %. **(F)** Quantification of LVEDD. **(G)** Quantification of LVESD. ANOVA, SNK test. EF: ejection fraction; FS: fraction shortening; LVEDD: left ventricular end diastolic diameter; LVESD: left ventricular end systolic diameter.

To confirm the above results, we evaluated infarct area by TTC staining on 1 dpMI.The results showed that the infarct area of the MI group was significantly larger than that of the sham group, while that of the MI + SF group was significantly smaller than that of the MI group (Figures 3A,B). As a result, on 7 dpMI, the EF% in the MI + SF group was significantly higher than that in the MI group (Figures 3C,D). Moreover, the increase in LVEDD and LVESD, as well as the decrease in FS%, were reversed by SF (Figures 3E–G). These results suggested that SF improved cardiac performance in the early period.

### 3.3 SF pretreatment reduces fibrotic production and increases cardiac performance in the late period

**FIGURE 4:**
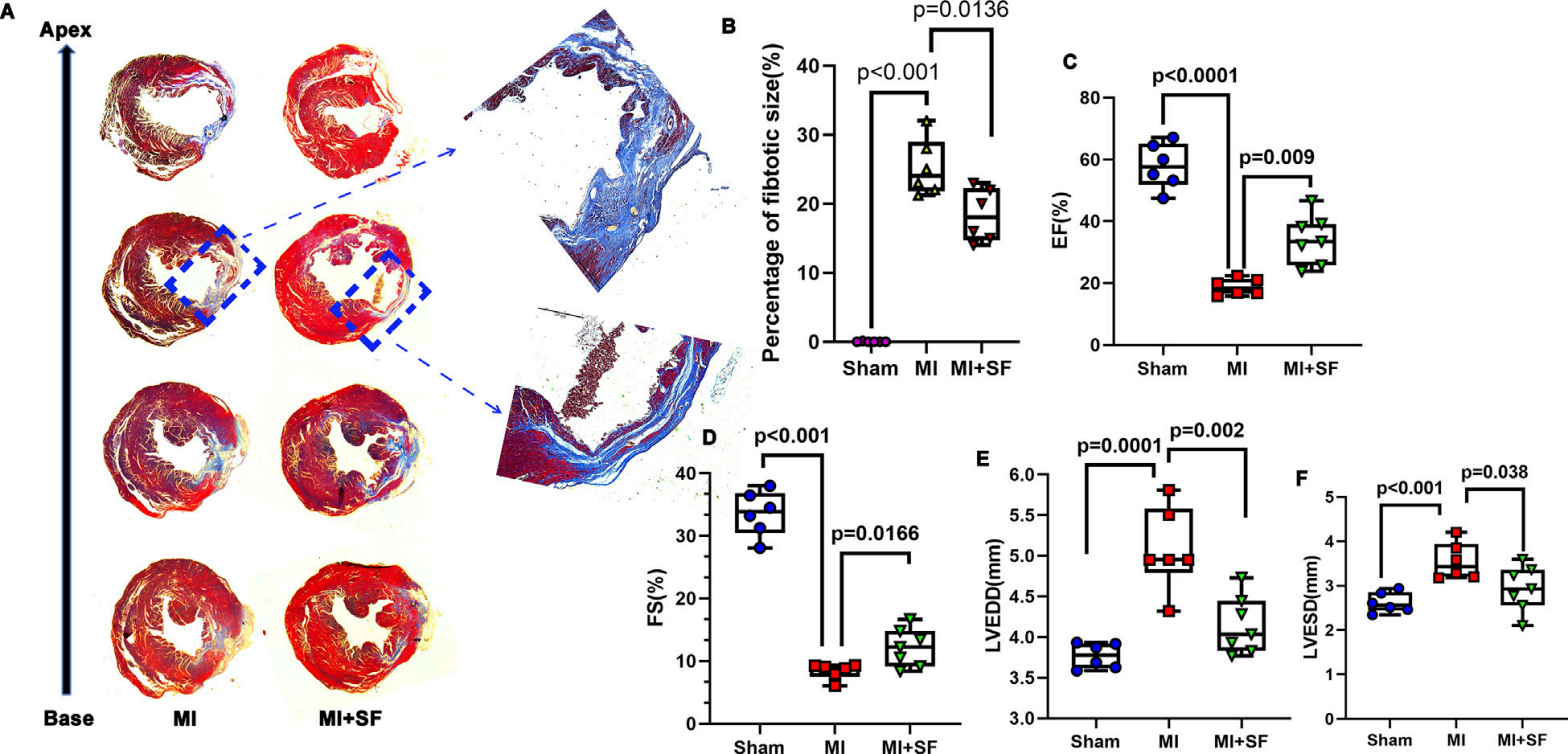
SF pretreatment reduces fibrosis production and increases cardiac function in the late period. **(A)** Representative fibrotic area visualized using Masson staining. **(B)** Quantification of fibrotic area (%). **(C)** Quantification of EF%. **(D)** Quantification of FS%. **(E)** Quantification of LVEDD. **(F)** Quantification of LVESD. ANOVA, SNK test. EF: ejection fraction; FS: fraction shortening; LVEDD: left ventricular end diastolic diameter; LVESD: left ventricular end systolic diameter.

To evaluate the long-term results of SF in MI protection, we evaluated fibrotic production by Masson’s trichrome staining on 28 dpMI. The results showed that the fibrotic area in the MI + SF group was significantly smaller than that of the MI group (Figures 4A,B). In addition, the EF% in the MI + SF group was significantly higher than that in the MI group (Figure 4C). Moreover, the decreased FS% and the increased LVEDD and LVESD caused by MI were reversed by SF (Figures 4D–F). These results suggested that SF improved cardiac performance in the late period.

### 3.4 SF pretreatment returned the gene expression profile of MI to normal (that of the sham) post-MI

**FIGURE 5:**
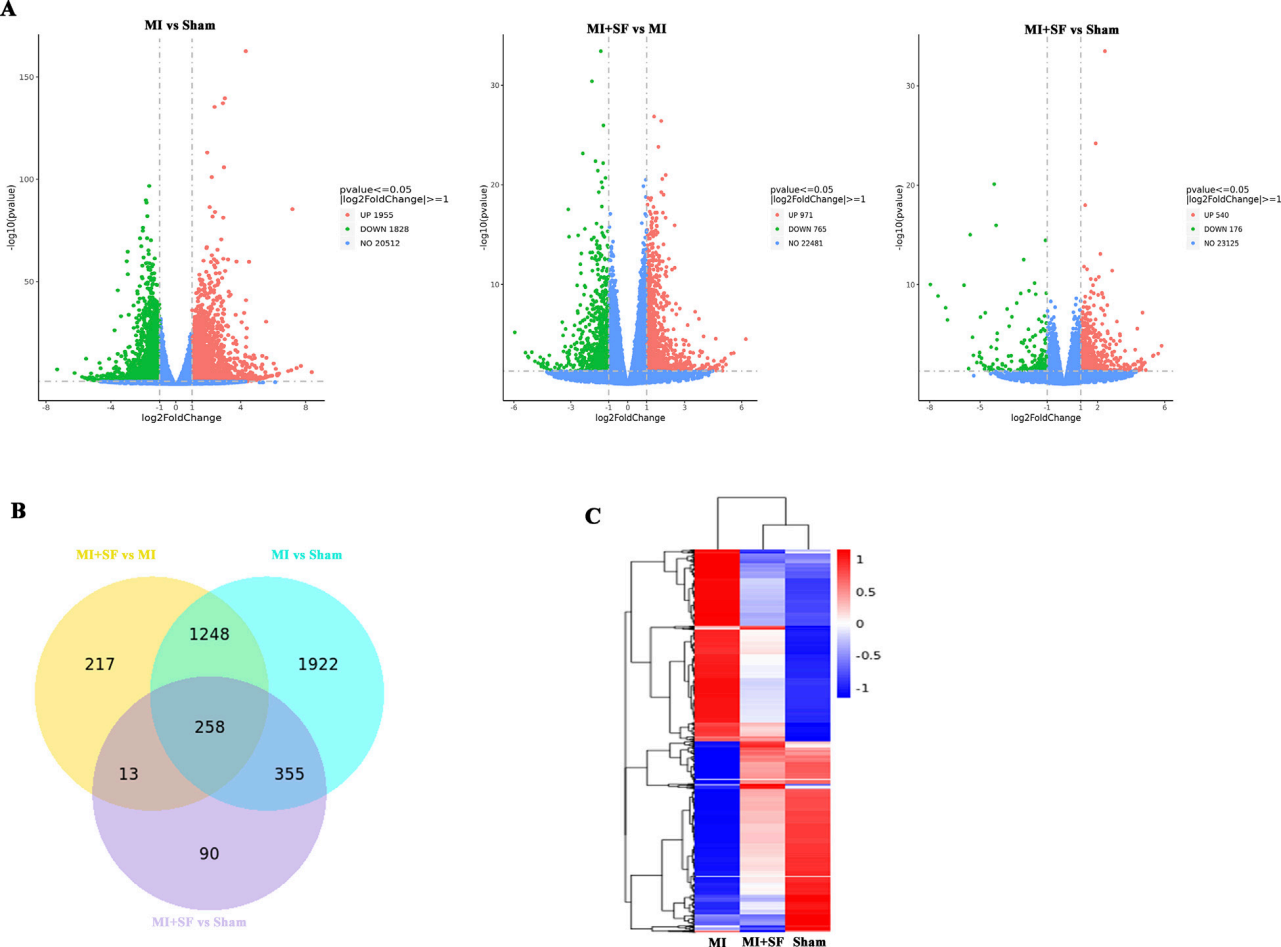
RNA seq revealed that SF pretreatment returned the gene expression profile of MI to normal (that of the sham) post-MI. **(A)** Volcano plot of differentially expressed genes (DEGs). Note that there were 3,783 DEGs between the MI and sham groups, with 1955 upregulated and 1828 downregulated; there were 1736 DEGs between the MISF and MI groups, with 971 upregulated and 765 downregulated; and there were 716 DEGs between the MISF and MI groups, with 540 upregulated and 176 downregulated. **(B)** Venn map of DEGs. **(C)** Heatmap of DEGs (3M vs. 1Y).

To understand why SF has a protective effect on MI, we performed RNA-seq analysis to investigate how the MI transcriptome was changed by SF. The volcano plots showed that there were 3,783 differentially expressed genes (DEGs) between the MI and sham groups, with 1955 upregulated and 1828 downregulated; 1736 DEGs between the MI + SF (MISF) and MI groups, with 971 upregulated and 765 downregulated; and 716 DEGs between the MISF and MI groups, with 540 upregulated and 176 downregulated (Figure 5A). The results suggested that SF reduced the number of DEGs caused by MI.

The Venn diagram shows the MISF vs MI groups and MI vs sham groups shared 1,248 + 258 = 1,508 common DEGs, the MISF vs MI groups and MISF vs sham groups shared 258 + 13 = 271 common DEGs, and the MI vs sham groups and MISF vs sham groups shared 258 + 335 = 593 common DEGs (Figure 4B). These results suggested that the MISF vs MI group comparison was more similar to the MI vs sham group comparison. The heatmap further confirmed the results, showing that the transcriptome in the MISF group was closer to that of the sham group (Figure 5C).

In summary, the above results showed that SF pretreatment returned the gene expression profile of MI to normal (that of the sham) post-MI.

### 3.5 GO enrichment analysis confirms SF’s protective effect post-MI and suggests a role of autophagy

**FIGURE 6:**
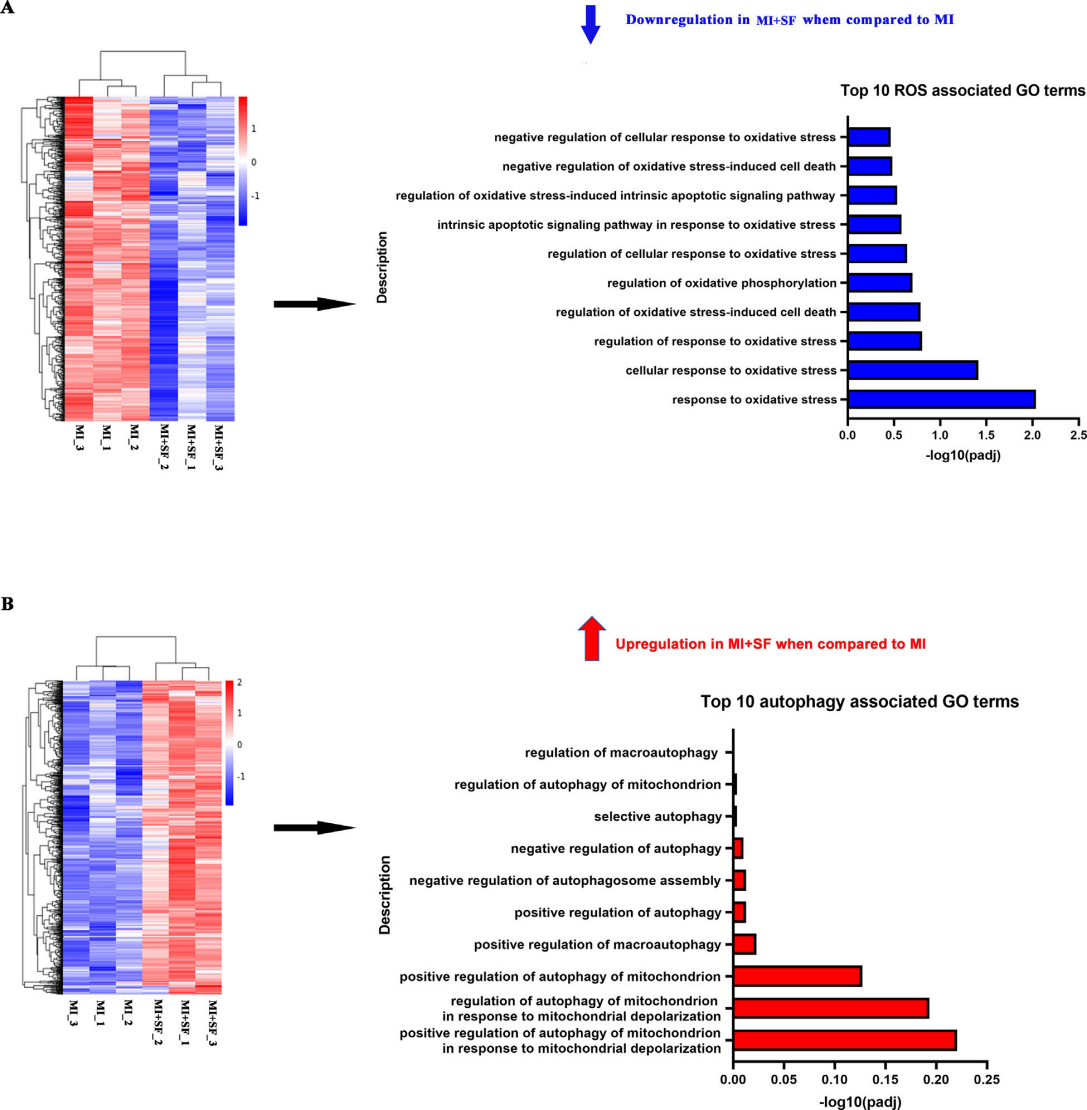
GO enrichment analysis revealed that SF pretreatment reduced the enrichment of ROS-associated genes while increasing the enrichment of autophagy-associated genes. **(A)** Top 10 ROS-associated GO terms of the downregulated DEG enrichment analysis. **(B)** Top 10 autophagy-associated GO terms of the upregulated DEG enrichment analysis.

Next, we conducted GO enrichment analysis on upregulated and downregulated genes separately (Figure 6; Supplementary Figure S1). The results showed that in the downregulated enrichment, there was an abundance of ROS-associated GO terms (Figure 6A) and inflammation- and apoptosis-associated GO terms (Supplementary Figure S1). These results suggest that SF has anti-inflammatory, anti-apoptotic, and antioxidant effects post-MI, consistent with previous results (Figures 2–4). Furthermore, in the upregulated enrichment, there was an abundance of autophagy-associated GO terms (Figure 6B), suggesting that autophagy may be one of the underlying mechanisms by which SF limits post-MI damage.

### 3.6 SF upregulates autophagic flux post-MI

**FIGURE 7:**
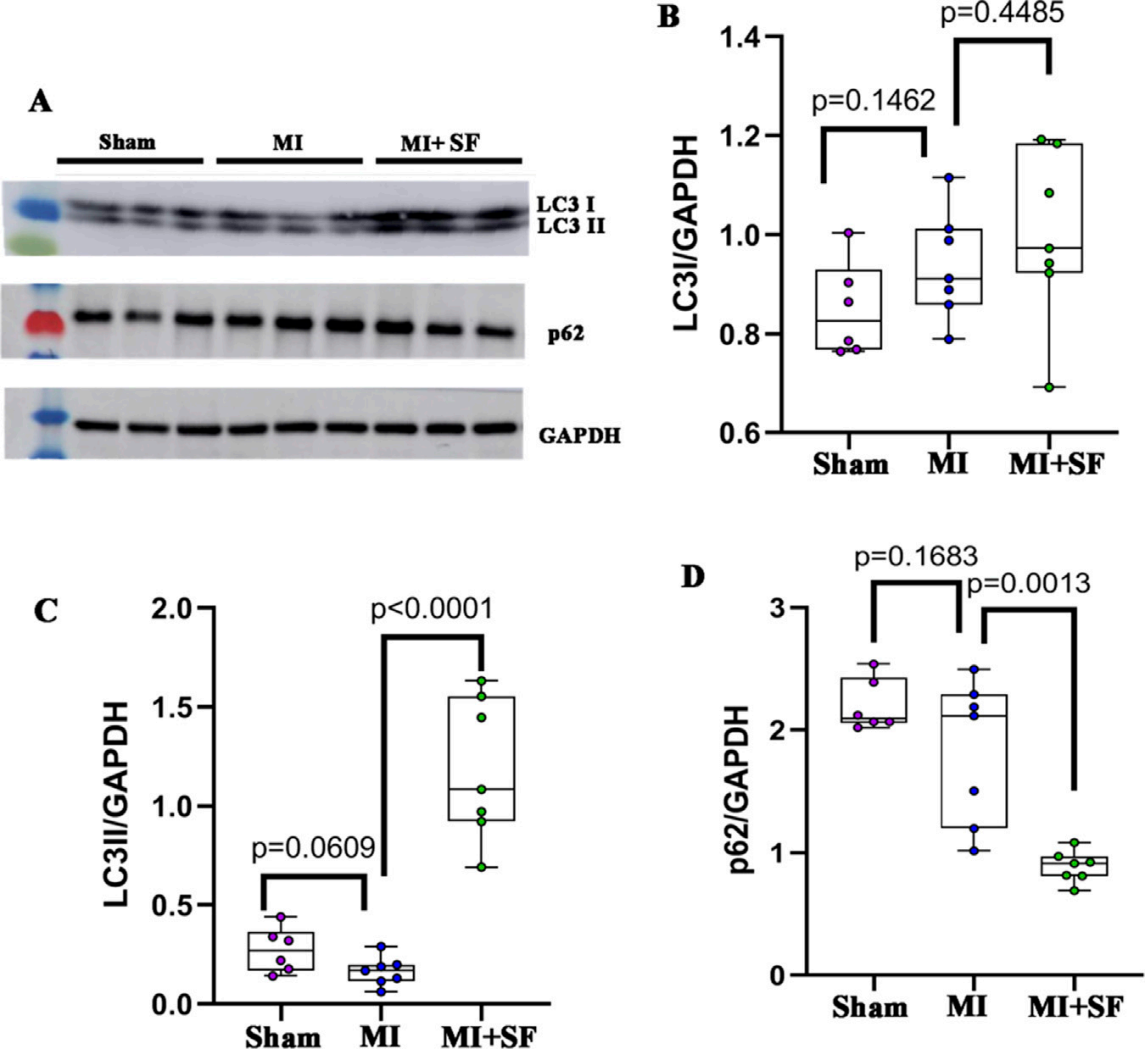
SF pretreatment promotes autophagic flux. **(A)** Representative blot of LC3I, LC3II, and p62. **(B)** Quantification of LC3I. **(C)** Quantification of LC3II. **(D)** Quantification of p62. ANOVA, SNK test.

To confirm the RNA-seq observation, we investigated whether SF induced an autophagic flux (Xiao et al., 2021). The results showed that LC3II in the MI + SF group increased significantly when compared with the MI group (Figures 7A–C), while p62 in the MI + SF group decreased significantly (Figures 7A,D). These results suggested that the autophagic flux was upregulated by SF post-MI.

### 3.7 Blocking autophagy inhibits the protective effect of SF post-MI

**FIGURE 8:**
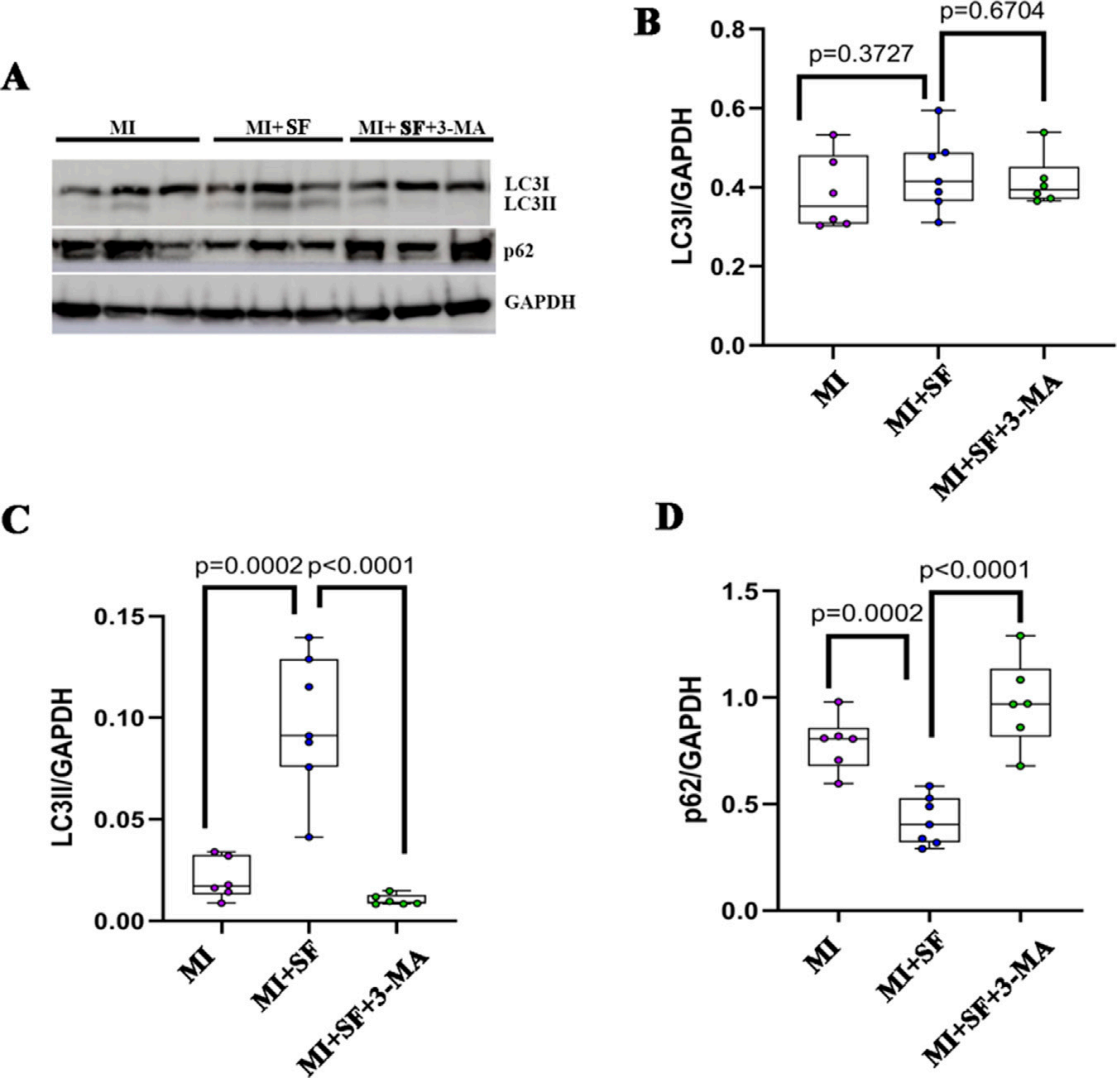
Autophagy inhibitor 3-MA blocks the autophagic flux induced by SF. **(A)** Representative blot of LC3I, LC3II, and p62. **(B)** Quantification of LC3I. **(C)** Quantification of LC3II. **(D)** Quantification of p62. ANOVA, SNK test.

To further confirm the role of autophagy in the protective effect of SF post-MI, we blocked the autophagic flux with 3-MA, a PI3 kinase (PI3K) inhibitor, which hinders the recruitment of LC3I to autophagosomal membranes (Xiao et al., 2021; Petiot et al., 2000). The results showed that the increase of LC3II expression by SF was blocked by 3-MA (Figures 8A–C). Consistent with the expression of LC3II, p62 also showed a reversed expression pattern (Figures 8A,D). These results indicated that 3-MA inhibited the upregulation of the autophagic flux induced by SF post-MI.

**FIGURE 9:**
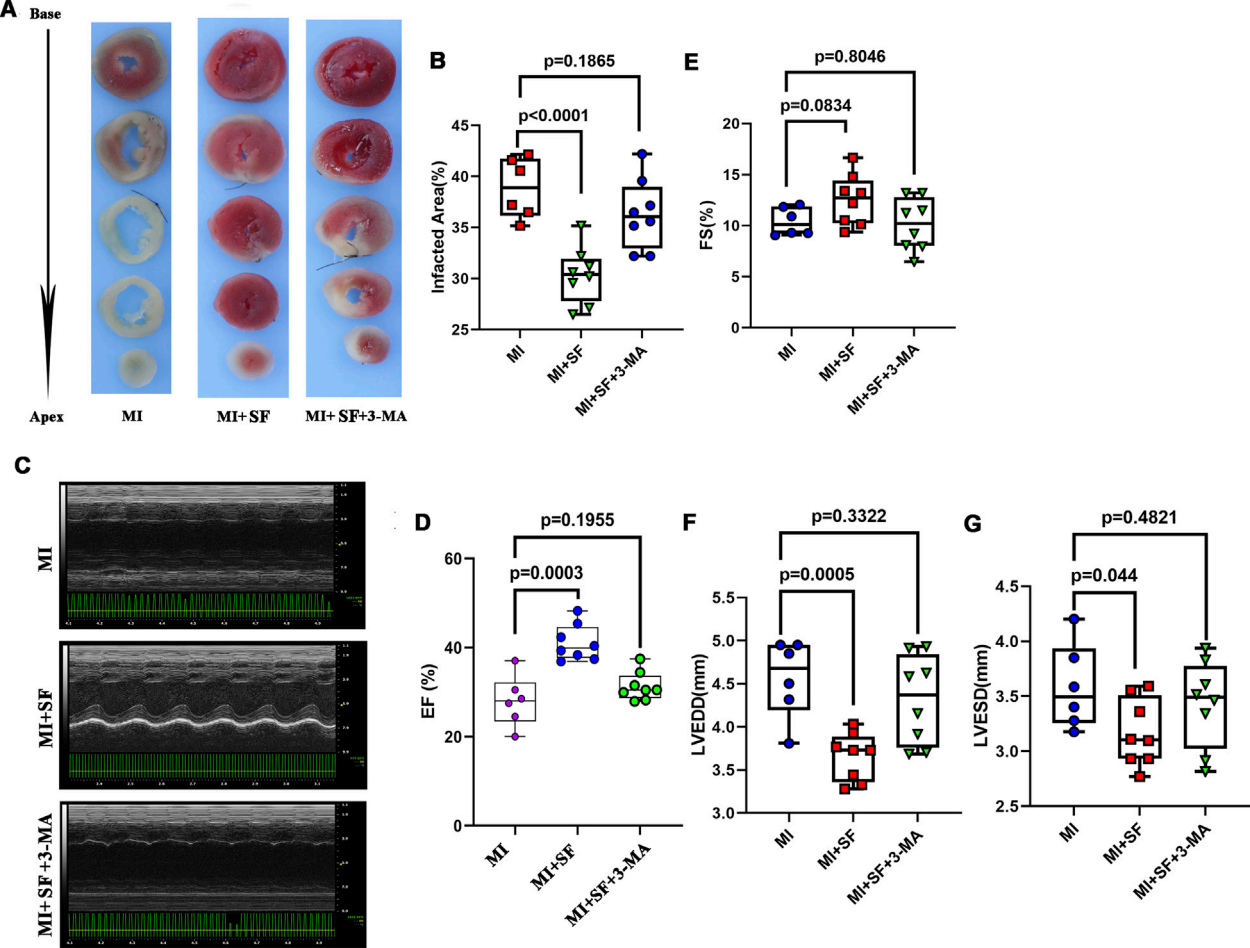
3-MA partially blocks the protective effect of SF post-MI. **(A)** Representative infarcted area by TTC staining. **(B)** Quantification of infarcted area (%). **(C)** Representative echocardiogram. **(D)** Quantification of EF%. ANOVA, SNK test. **(E)** Quantification of FS %. **(F)** Quantification of LVEDD. **(G)** Quantification of LVESD. EF: ejection fraction; FS: fraction shortening; LVEDD: left ventricular end diastolic diameter; LVESD: left ventricular end systolic diameter.

As a result, the reduced infarcted area was reversed by 3-MA in the MI + SF+3-MA group (Figures 9A,B), and the improved cardiac performance resulting from SF was preserved by 3-MA in the MI + SF+3-MA group (Figures 9C–G). These results suggested that the protective effect exerted by SF post-MI depended, at least in part, on autophagy.

## 4 Discussion

Electrolyte solutions are widely used in clinical treatment with great therapeutic effects due to their powerful ability to compensate for fluctuations in the internal environment, especially in intensive care, surgery, and infectious diseases (Ellekjaer et al., 2020; Lavoie and Chessex, 2019; Brooke et al., 1962; Feng et al., 2023; Sankar et al., 2023). Despite extensive experience in the use of electrolyte solutions, constant progress in electrolyte solutions continues with new ionic formulas being developed (Ellekjaer et al., 2020; Lavoie and Chessex, 2019; Brooke et al., 1962; Feng et al., 2023; Sankar et al., 2023). In addition to maintaining consistency with the plasma composition as much as possible, new metabolizable organic anions (e.g., acetate, malate, lactate) are added to enhance the acid-base, ionic, and metabolic balance capabilities of the electrolyte solution (Ellekjaer et al., 2020; Lavoie and Chessex, 2019; Brooke et al., 1962; Feng et al., 2023; Sankar et al., 2023). Deepening the understanding of the efficacy and mechanisms of electrolyte solutions, in particular situations, is undoubtedly crucial for continuous progress in this field.

In this study, we first demonstrated that SF, a widely used electrolyte solution in clinical settings, had anti-inflammatory, antioxidant, and anti-apoptotic effects post-MI. ROS is an important cause of cell death after MI (Wang et al., 2018; Akodad et al., 2020; Cai et al., 2023). SF may clear ROS through three pathways. Firstly, SF can upregulate the expression of oxygen free radical scavengers such as superoxide dismutase (SOD) and catalase (CAT) by its component, malate (Zeng et al., 2015). Secondly, malate can directly act as a precursor of succinic acid to improve mitochondrial metabolism efficiency (Nielsen et al., 2011). Thirdly, autophagy is an effective means of clearing ROS (Kanki et al., 2011), as it also evidences in current study. How SF clear ROS values further investigation.

In addition, we demonstrated that SF’s cardiac protection were associated with the upregulation of autophagy. Thus, the current study expanded our understanding of the functions and mechanisms of electrolyte solutions, potentially contributing to the progress of the electrolyte solution field and intensive care. However, autophagy is dependent on the acid-base status and ATP supply (Song et al., 2018; Barnaba et al., 2023; Song et al., 2023; Cui et al., 2023). Further in-depth research is needed to determine whether SF plays a direct role in regulating autophagy or whether its autophagy modification is secondary to its acid-base balance or ATP regulation. In addition, it is unclear whether there are other mechanisms involved in the protective effect of SF post-MI in addition to autophagy. In our RNA-seq results, the top 30 enriched GO terms of upregulated genes were mainly associated with ion transport, especially potassium transport (Supplementary Figure S2). It is possible that potassium transport is a foundation of SF-mediated protection against MI, because MI, SF, and autophagy converge on potassium (Zadák et al., 2010; Sharma et al., 2023; Wang X. et al., 2023).

**FIGURE 10:**
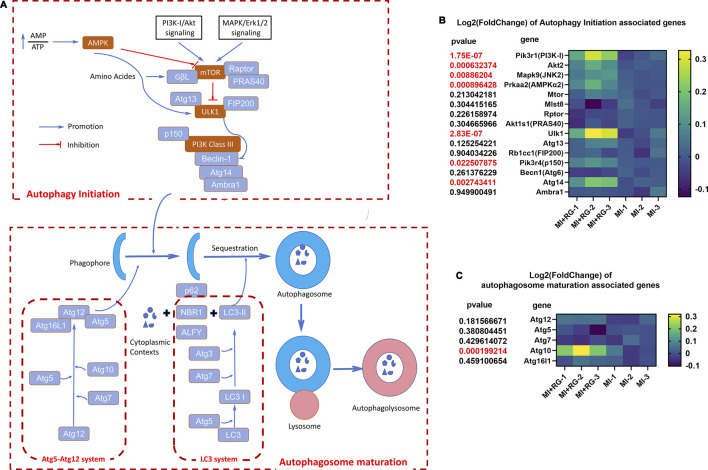
Autophagy signaling pathway and changes in autophagy-associated genes induced by SF. **(A)** Autophagy signaling pathway and its associated molecules. **(B)** Heatmap of autophagy initiation-associated genes. **(C)** Heatmap of autophagosome maturation-associated genes.

What is next in investigating the specific pathways by which SF regulates autophagy? Based on recent publications (Wang L. et al., 2023; Debnath et al., 2023), we illustrated the autophagy signaling pathway (Figure 10A) to better understand the possible underlying mechanisms of SF’s MI protection; our data demonstrated that, to initiate autophagy, AMPK signaling, which was found to be regulated by AMP/ATP balance, might be involved in SF’s MI protection. Moreover, the downstream key effector ULK-1 might be increased by SF (Figure 10B); to improve the autophagosome maturation, SF also increased the expression of Atg 10 (Figure 10C). In conclusion, these data suggest that both autophagy initiation and autophagosome maturation might be regulated by SF to protect MI. Further investigations could focus on the specific autophagy molecules to fully understand how SF pretreatment prevents MI-induced heart damage.

One limitation of this study is that we pretreated mice with SF before MI occurred, and it is unclear whether injecting SF after MI still has similar effects. In clinical practice, we usually use electrolytes as treatment after MI and rarely use electrolytes for prevention before MI occurs. Treatment and prevention belong to two different research categories involving different mechanisms. Prevention is relatively simple, while treatment is often more complex, requiring the determination of treatment dosage and duration, the evaluation of short-term and long-term effects, involving more complex mechanisms such as long-term fibrosis and myocardial regeneration. Further research is needed to determine whether and how SF treatment after MI still protects the heart from MI damage.

Another limitation is the presence of potential confounders that could influence the results, such as SF and 3-MA vehicles. To address these two confounders, we compared the SF group to the sham group and the 3-MA vehicle + MI group to the MI group. The data showed that SF had no effects on ROS production, infarcted area, cardiac performance, and autophagic flux when compared to the sham group (Supplementary Figures S3–S5), and that the vehicle had no effect on the infarcted area or autophagic flux (Supplementary Figures S6–S7). In addition, all surgeries and measurements were performed under the same standardized conditions.

In summary, the current study demonstrated that in a post-MI situation, pretreatment with the electrolyte solution SF protected mice from post-MI damage, and the underlying mechanisms were associated with the upregulation of autophagy, thus deepening our understanding of electrolyte solutions in clinical use. However, there are several questions that remain. For example, in addition to autophagy, is there any other mechanism underlying SF-mediated protection against MI? Is autophagy modification by SF direct or secondary to its acid-base balance regulation? In addition to MI, does SF show anti-inflammatory, antioxidant, or anti-apoptotic activity in other clinical settings, such as surgery or infectious diseases? In addition to SF, can other electrolyte solutions show similar protective effects? Answering these questions will contribute to continuous progress in the field of electrolyte solutions.

## Data Availability

The original contributions presented in the study are publicly available. This data can be found at the NCBI’s Gene Expression Omnibus database, accession number GSE245917.
