# Correction: Cardioprotective effects of the electrolyte solution sterofundin and the possible underlying mechanisms

**DOI:** 10.3389/fphar.2025.1633008

**Published:** 2025-06-09

**Authors:** Min Chen, Yingying Xiao, Jijian Zheng, Peibin Zhao, Lin Cheng, Chuan Jiang, Sixie Zheng, Zheng Wang, Sijuan Sun, Lincai Ye, Guozhen Chen, Hao Zhang, Yanhui Huang

**Affiliations:** ^1^ Children’s Heart Center, Institute of Cardiovascular Development and Translational Medicine, The Second Affiliated Hospital and Yuying Children’s Hospital, Wenzhou Medical University, Wenzhou, China; ^2^ Department of Anesthesiology, Shanghai Children’s Medical Center, Shanghai Jiao Tong University School of Medicine, Shanghai, China; ^3^ Department of Thoracic and Cardiovascular Surgery, Shanghai Children’s Hospital, Shanghai Jiao Tong University School of Medicine, Shanghai, China; ^4^ Department of Thoracic and Cardiovascular Surgery, Shanghai Children’s Medical Center, Shanghai Jiao Tong University School of Medicine, Shanghai, China; ^5^ Department of Pediatric Intensive Care Unit, Shanghai Children’s Medical Center, Shanghai Jiao Tong University School of Medicine, Shanghai, China; ^6^ Shanghai Institute For Pediatric Congenital Heart Disease, Shanghai Children’s Medical Center, School of Medicine Shanghai Jiao Tong University, Shanghai, China; ^7^ Department of Cardiology, Shanghai Children’s Medical Center, Shanghai Jiao Tong University School of Medicine, Shanghai, China

**Keywords:** sterofundin, electrolyte solutions, myocardium infarction, intensive care, autophagy

In the published article, there was an error in the affiliations. The order of affiliations 1 and 2 was inverted.

The authors apologize for this error and state that this does not change the scientific conclusions of the article in any way. The original article has been updated.

