# Supplementary material for: Cardioprotective effects of the electrolyte solution sterofundin and the possible underlying mechanisms
Source: Front Pharmacol. 2025 Jan 3;15:1449831. doi: 10.3389/fphar.2024.1449831 (PMC11738938; doi:10.3389/fphar.2024.1449831)
Supplement: Supplementary file 1 [file DataSheet2.pdf]

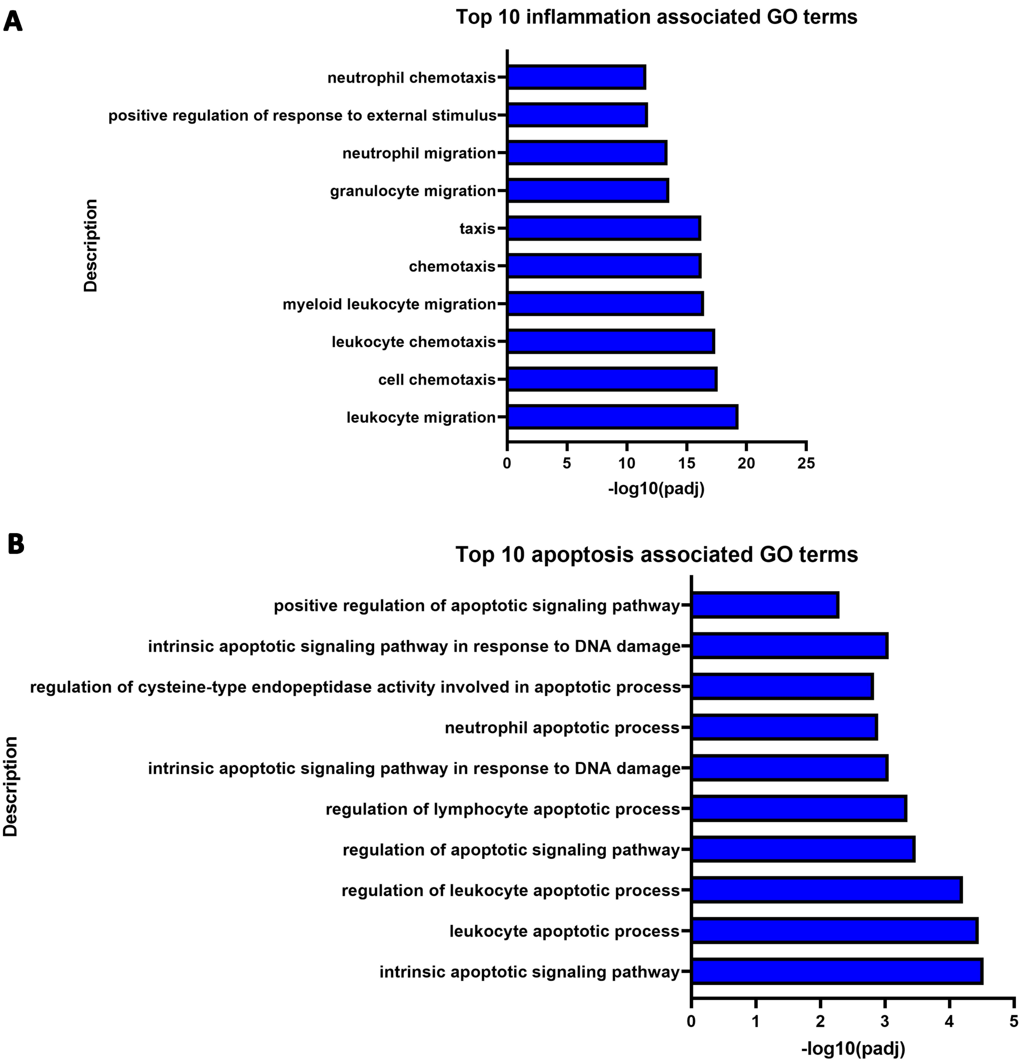

Supplemental Fig. S1 GO enrichment analysis revealed that SF pretreatment reduced the enrichment of inflammation- and apoptosis-associated genes. (A) Top 10 inflammation-associated GO terms of the downregulated DEG enrichment analysis. (B) Top 10 apoptosis-associated GO terms of the downregulated DEG enrichment analysis.

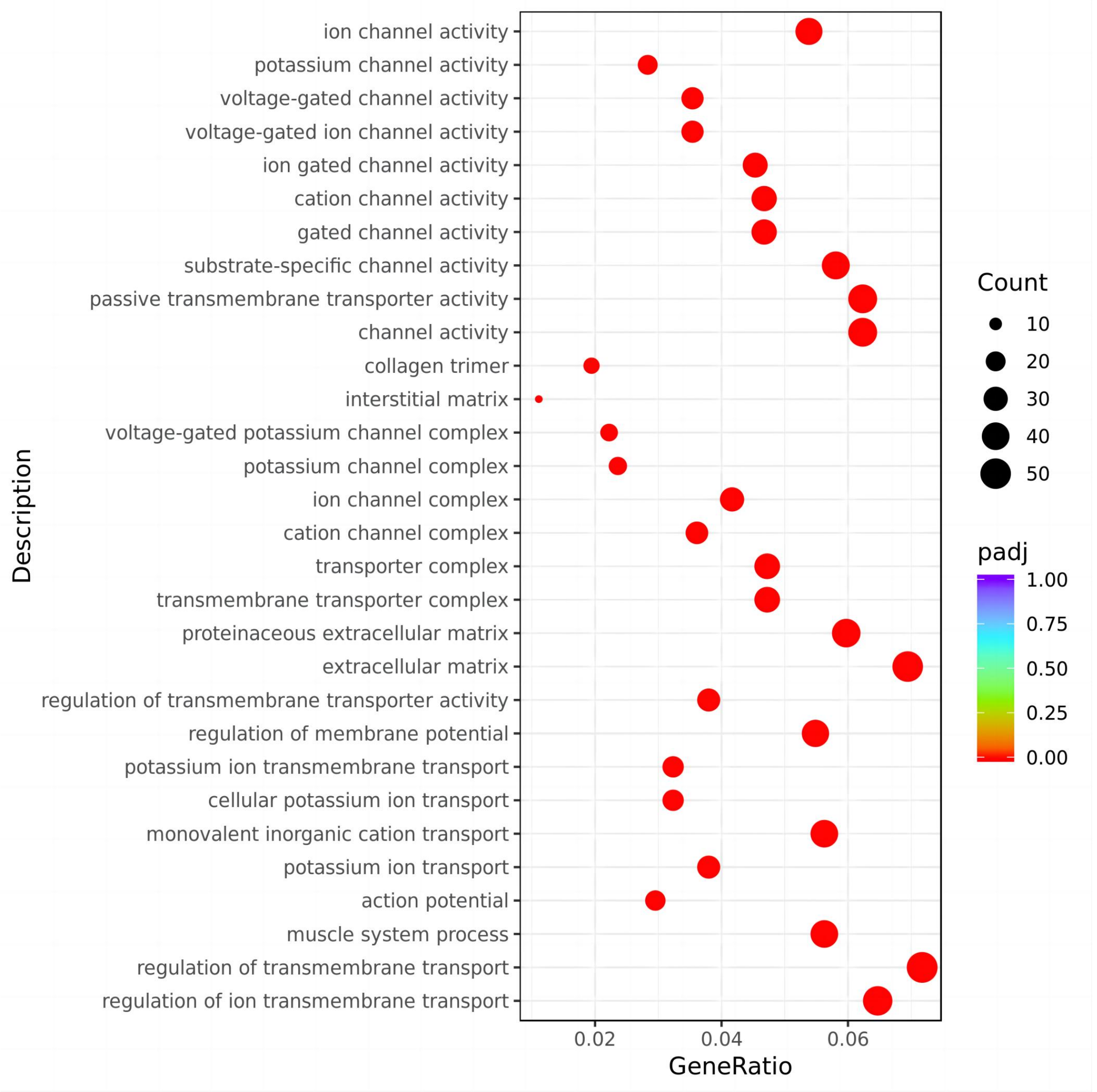

Supplemental Fig. S2 Top 30 enriched GO terms of upregulated genes.

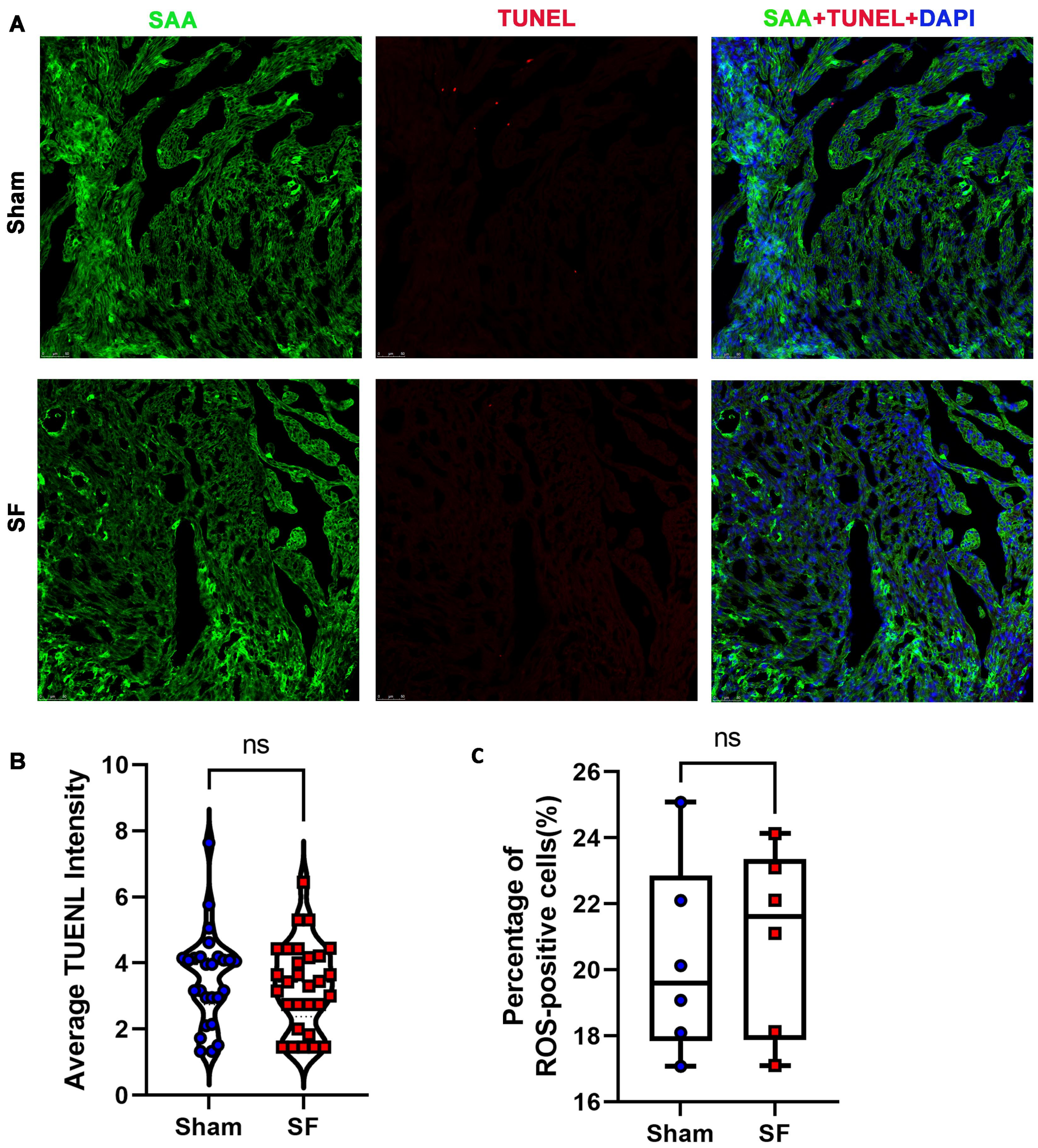

Supplemental Fig. S3 SF have no effects on ROS and cell necrosis. (A) Representative TUNEL-positive cells. SAA (Sarcometric α actinin, marker of cardiomyocyte, green), DAPI (blue), and TUNEL (red). (B) Quantification of TUNEL intensity. (C) Quantification of ROS-positive cells. Student t- test.

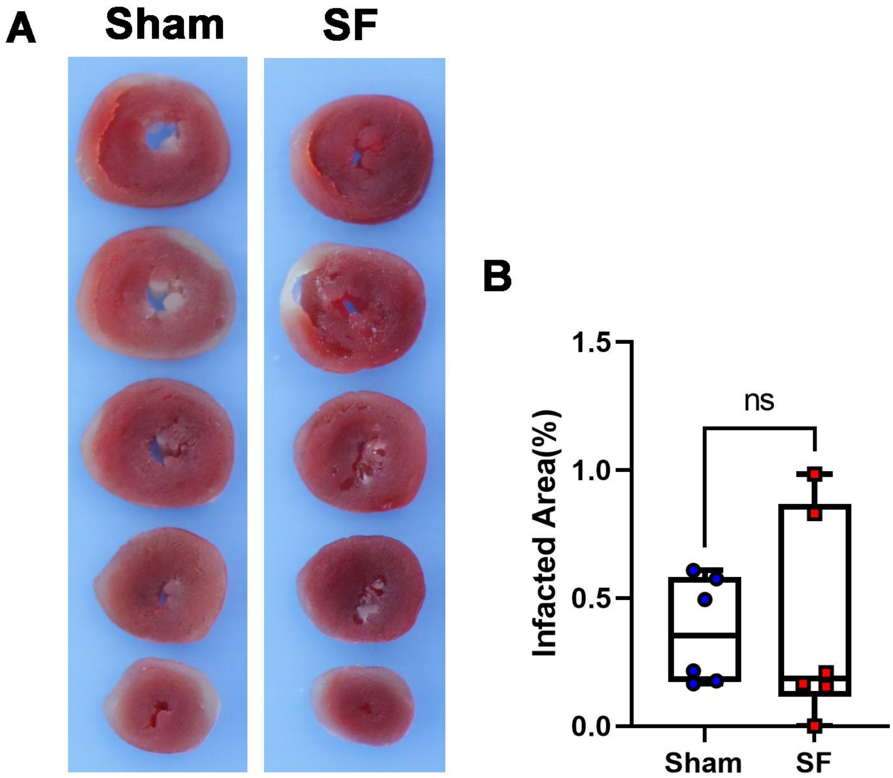

Supplemental Fig. S4 SF has no effect on infarct size at early period. (A) Representative infarcted area by TTC staining. (B) Quantification of infarcted area (%).

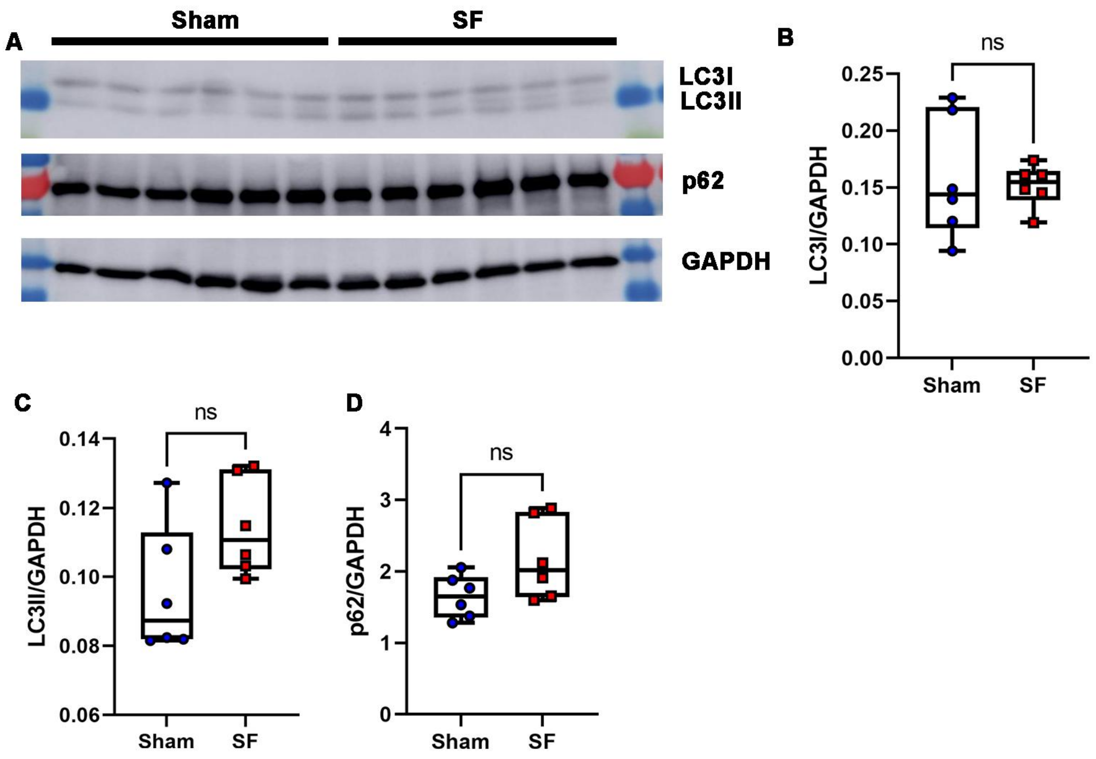

Supplemental Fig. S5 SF has no effect on autophagic flux. (A) Representative blot of LC3I, LC3II, and p62. (B) Quantification of LC3I. (C) Quantification of LC3II. (D) Quantification of p62.Student t-test

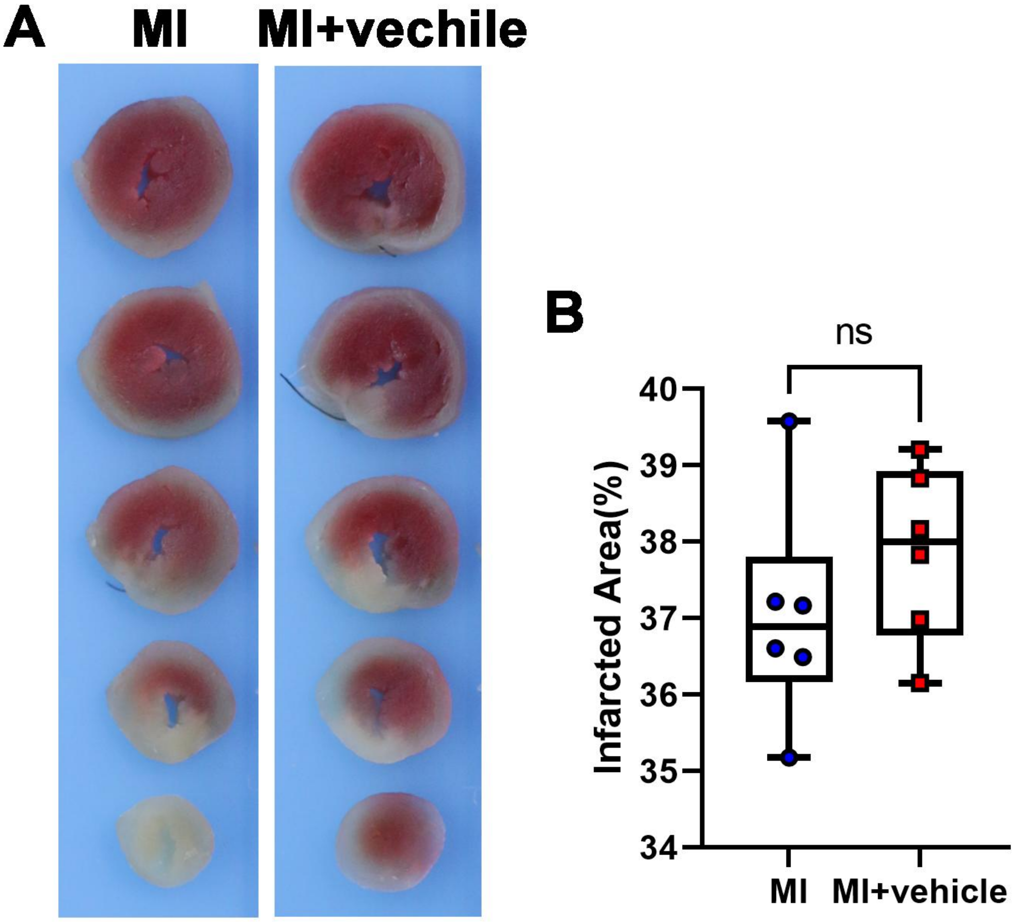

Supplemental Fig. S6 3-MA vehicle (DMSO) has no effect on infarct size at early period. (A) Representative infarcted area by TTC staining. (B) Quantification of infarcted area (%).

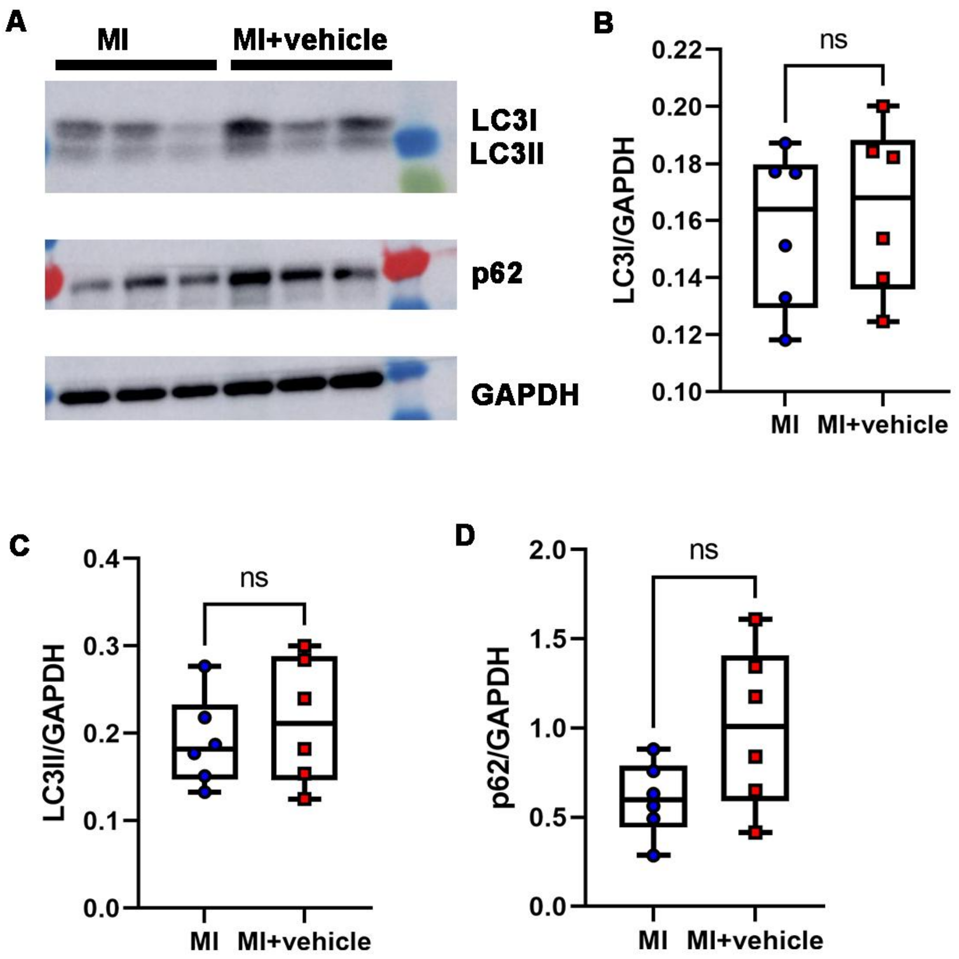

Supplemental Fig. S7 3-MA vehicle (DMSO) has no effect on autophagic flux. (A) Representative blot of LC3I, LC3II, and p62. (B) Quantification of LC3I. (C) Quantification of LC3II. (D) Quantification of p62. Student *t*-test
