# Supplementary material for: Cardioprotective effects of the electrolyte solution sterofundin and the possible underlying mechanisms
Source: Front Pharmacol. 2025 Jan 3;15:1449831. doi: 10.3389/fphar.2024.1449831 (PMC11738938; doi:10.3389/fphar.2024.1449831)
Supplement: Supplementary file 2 [file DataSheet1.pdf]

Mouse tail vein injection

For experienced technicians, one injection would take him or her less than 5 minutes. With 6 technicians(including trained students), 30 min is far enough for technicians to complete 36 injections. A mouse has three different tail veins that can be used for injection in turn. However, it should be noted that the injection site should start from the distal end and then move towards the proximal end.

Please refer to ucdavis's teil vein 's injection protocol(https://cmgi.ucdavis.edu/files/2015/01/Mouse_Tail_Vein_Catheter_Procedure_Rev2.pdf).

The following is the mouse tail Vein injection protocol. Although the protocol suggests that the dorsal tail vein is difficult to access and not for tail vein injection, in our experiences, it can still be served as a backup for injection.

Intravenous injection into the mouse lateral tail vein can be a challenging procedure. However, utilization of a tail vein catheter can greatly improve the likelihood of a successful IV injection. We have used the following procedures with success in the CMGI (Center for Molecular and Genomic Imaging; U.C. Davis), and currently use a tail vein catheter for more than 95% of our mouse intravenous injections.

Mouse Tail Anatomy

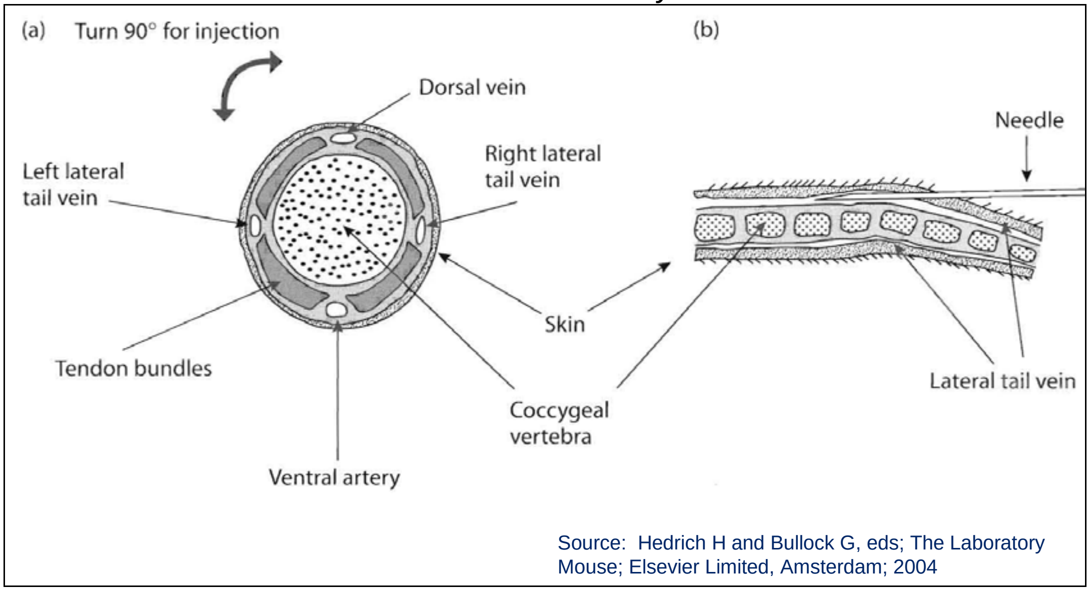

The two lateral tail veins are small vessels on each side of the tail, directly under the skin. There is also a dorsal tail vein, but it's more difficult to access and is not used for tail vein injection.

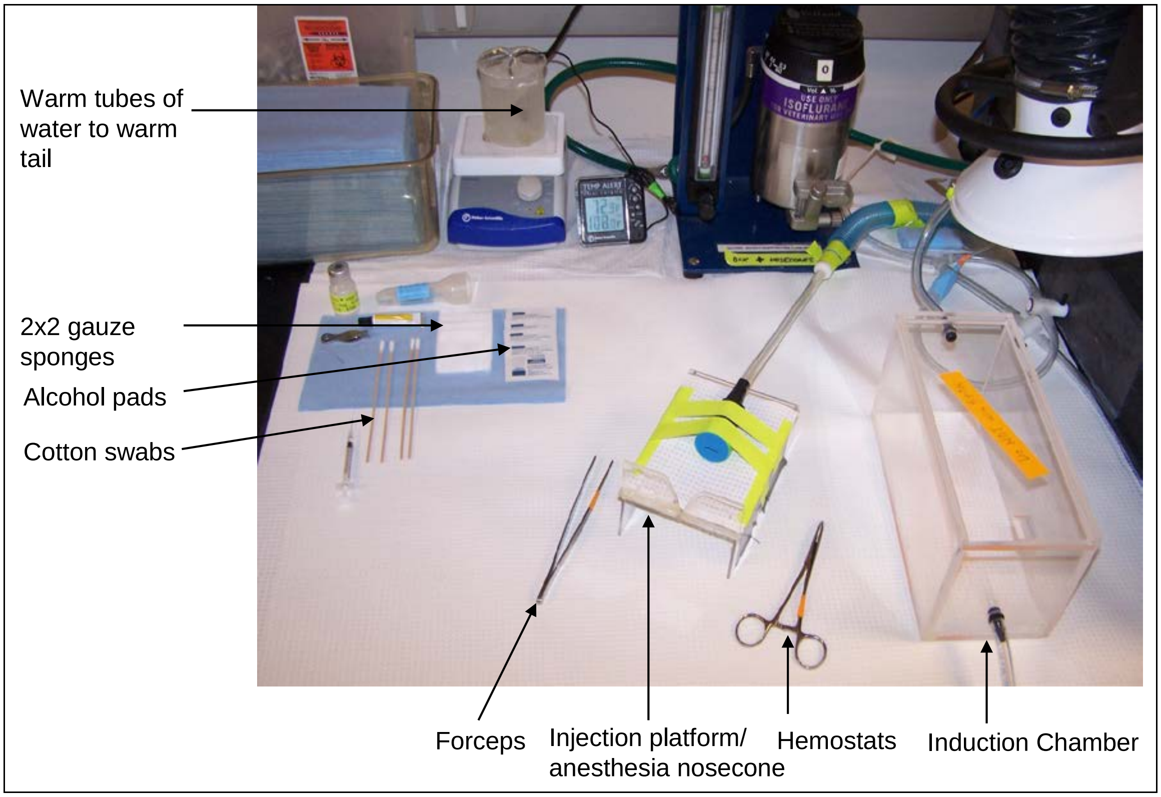

The injection station shown above displays the supplies and equipment we typically use for tail vein catheterization in the isoflurane-anesthetized mouse. The injection platform is laboratory-built (Plexiglas), and has a slot at the back to pull the tail through to maintain tension during needle insertion. For some studies we use a commercially available injection platform/tail warmer (Braintree Scientific), which has a built-in heat source to help keep the animal warm. To maintain the animal's body temperature, we use a heat lamp when the mouse is in the induction chamber and on the lab-built injection platform.

Preparation of catheters

The catheters we use are made in our laboratory, and we've found several different sizes to work well:

1. PE10 tubing (~3" length) with 30 ga needle
2. Microrenathane (MRE-025; Braintree Scientific) with 28 ga needle
3. PE20 tubing with 25 ga needle

To make the catheter, the needle is repeatedly bent back and forth until it breaks from the hub. The blunt end is then pushed into the end of the catheter tubing. It helps to hold the needle with a pair of hemostats or forceps. We usually use the 30 ga catheter and use a ½ cc insulin syringe w/ 28 ga x ½" needle for injection into the catheter.

Catheter insertion procedure

Prepare the catheter for insertion by preloading it with heparinized saline (0.5 ml heparin [1,000 USP units/ml] in 10 ml of sterile saline). Position the anesthetized mouse on its side so the lateral tail vein is uppermost. Clean the tail with a 70% alcohol prep pad. One of the keys to successful catheter insertion is to have the animal, particularly the tail, warmed to dilate the vein, which may be partially constricted due to the anesthetic. It can be helpful to place the tail in a small plastic tube of warm water (105° – 115°F). Equally important is to match the angle of the needle with the angle of the vein. To achieve this, the tail is bent slightly so the approach angle of the needle is almost parallel to the vein (pictured below). Use the dominant hand to hold the catheter, the other to grasp the tail between the thumb and index finger, thumb on top, then pull back slightly so the tail is firm. The catheter needle is held with a hemostat or forceps and the needle, bevel up, is introduced through the skin at a flat angle and advanced for several mm. Blood will usually enter the catheter if the needle is in the vein. If no blood enters the catheter but you believe it was inserted correctly, using slight pressure with your thumb and sweeping back along the tail will flush blood into the catheter if it is properly inserted. Also, to verify correct insertion and that the vein is not perforated, use a small saline flush into the catheter. If the needle is in the vein, the plunger moves easily and the dark colored vein clears as the saline is injected. If the plunger is hard to push or the tail around the injection site blanches white, the needle tip is not in the vein, so the needle should be withdrawn. If needed, secure the catheter in place with tape (eg, transpore tape). It is critical to keep the needle in the same position once in the vein so it doesn't poke through the vessel wall. Because there is always the possibility that the insertion will be unsuccessful, the first attempt should be made in the end third of the tail, so that subsequent tries can be done on parts of the tail closer to the body, downstream from previous puncture sites.

Some investigators find it useful to temporarily occlude the vein with a rubber band or piece of suture material at the base of the tail, to block the flow of blood back to the body and thereby swell the tail vein. Take care not to occlude so tightly that arterial blood flow is also blocked. Once the needle is inserted, the occluder is removed before infusion.

After the catheter is removed, apply pressure with gauze or a cotton swab for 30-60 sec or until bleeding stops.

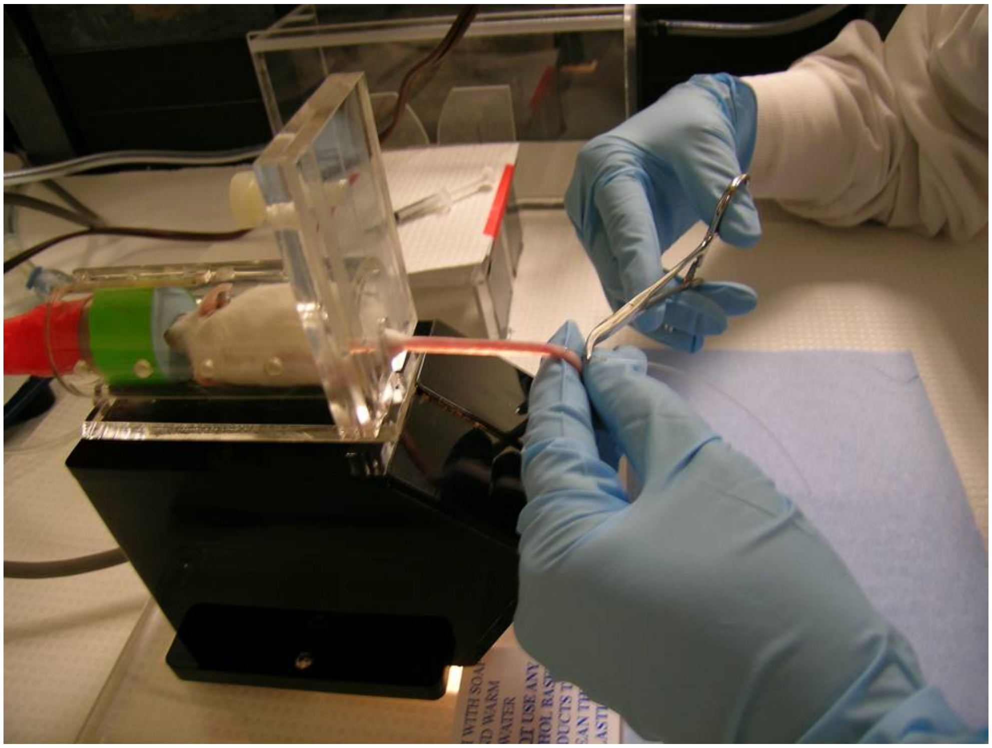

NOTE: Animal is on its side and the tail is bent and pulled back so the needle can be inserted at a flat angle into the vein.
